# Public Schools

**Published:** 1860-06

**Authors:** 


### PUBLIC SCHOOLS.

THE May article on this subject has elicited very considerable attention and commendation. A physician of long experience and close observation called to express his gratification, and, at our request, made the following statement corroborative of our views:

"I have, in many instances, explained, warned, and remonstrated in vain with parents, against the overstraining of the brains of children. A lady informed me that she did not know what to do with her little boy, aged six years. He had become fractious and irritable; small provocations threw him into a violent passion, and he was becoming uncontrollable. On inquiry, I learned he was going to school, was learning very fast, often repeated parts of his lesson in his sleep, which was restless and disturbed. At one time his head was hot, at another the perspiration from it would drench his pillow; his appetite was capricious, his limbs were small, his flesh, which he was losing, was soft; all indicating, as I informed her, an over-worked brain, and that the powers of life were being rapidly exhausted. She promised to speak to the father, but proceeded: 'What in the world shall I do with him? He is so fractious, there is no living in the same house with him. Besides, the teachers do not want to give him up. He is one of the best scholars at school. If taken away now, reädmission may be refused hereafter.' In about a month after I met the mother again, but she was in deep mourning. The element of household disturbance had been removed, but in a manner most unwelcome. The child had taken a fever, which at once settled on the brain. The 'best boy in the school' talking incessantly: day and night, in his delirium, he kept on repeating parts of his lessons, and so continued until the last hour of his life!

"This is not a solitary case. There are others like it of daily occurrence, and the child of promise and of pride passes away; the parents, meanwhile, all unconscious of the very direct agency they had in a death so premature and sad. Prompt reforms are needed in all schools, public and private; parents and people, the pulpit and the press, should agitate and agitate until wiser and better and more humane counsels prevail. Our public schools are too much on the high pressure system; too many hours are allotted to study; too many lessons; too many kinds at one time; too much urging; too much stimulation by compulsion or penalty; by shame or fear; above all, too much is imposed on the pupils out of school-hours. Mothers in feeble health are compelled to toil from morning until night in the performance of household duties, while their half-grown daughters are poring over their hard lessons, with tired and fevered brains; both mother and children needing help in their respective employments."

To these well-put statements of Dr. Shepherd, another may be added, which commends itself to the humane. Public schools were primarily intended for the poor, whose children otherwise would have grown up in ignorance, neglect, idleness, and crime. But by the systematic, thorough, and able manner in which they have been conducted in the city of New-York, some of them, especially those located in certain districts, have so commended themselves to the higher classes, that, in bad weather, private carriages, with their various liveries, are seen to take up or deposit the "responsibilities" of our wealthier citizens at the doors of the public school-room, with this most oppressive result to the poor, and their children: Lessons are invariably given to the children to be learned at home, in addition to the six hours spent in the school-room; to master these requires almost the entire time from dismission in the afternoon until the hour for reässembling in the morning; and even this would, in many instances, be an impossible task but for the aid which educated and well-to-do parents can render their children. But when the parents are poor and ignorant, they have not an hour to spare, even if they had the mental ability to aid their children in their tasks. In fact, in many cases, the almost discouraged and exhausted mother needs the aid of her half-grown daughters, as Dr. Shepherd so well observed, in the performance of indispensable household duties. How many of these children of poverty, in their ambition to stand well in their classes, study themselves into exhausting nervous fevers, inflammation of the brain, and an early death, we may never know, but the number must be great! This goading of the children of the poor to death is worse than the overseer's lash; it is literally a murderous inhumanity, and reason and common-sense call aloud for its immediate rectification, by the unconditional and absolute abolishment of study out of school-hours, in the city, out of the city, every where!

